# Coexistence of MRCS syndrome, extremely long axis and exfoliation syndrome: a case report and literature review

**DOI:** 10.1186/s12886-023-02965-7

**Published:** 2023-05-30

**Authors:** Xinglin Wang, Xiaodan Jiang, Ziyuan Liu, Changguan Wang, Xuemin Li

**Affiliations:** 1 Department of Ophthalmology, Peking University Third Hospital, Haidian District, 49 North Garden Road, Beijing, China; 2 Beijing Key Laboratory of Restoration of Damaged Ocular Nerve, Haidian District, 49 North Garden Road, Beijing, China

**Keywords:** MRCS syndrome, Exfoliation syndrome, Extremely long axial length, Case report

## Abstract

**Background:**

The coexistence of MRCS (microcornea, retinal dystrophy, cataract, and posterior staphyloma) syndrome and extremely long axis is rare since microcornea frequently accompanies with diminution of entire anterior segment and occasionally the whole globe. In the case presented here, combination of these two elements were identified, together with XFS (exfoliation syndrome).

**Case presentation:**

A 66-year-old Han Chinese woman presented for consultation due to impaired vision which accompanied throughout her entire life span and worsened during the last 2 years. Combination of MRCS syndrome and extremely long axial length was evidently diagnosed in both eyes, with XFS confirmed in her right eye, but mutation screening failed to identify disease-causing sequence variants in some specific genes reported previously, including BEST1 and ARL2. However, likely pathogenic mutations in FBN2 gene were identified. Bilateral cataract phacoemulsification without intraocular lens implantation was performed using scleral tunnel incision and under general anesthesia. At 3-month follow-up, ocular recovery of the patient was satisfactory.

**Conclusions:**

The case presented here exhibited rare coexistence of MRCS syndrome, extremely long axis and XFS. The complexity of her ocular abnormalities brought challenges to surgical management, in which multidisciplinary collaboration is often required. Furthermore, the genetic analysis in this case yielded a possible novel candidate gene for MRCS syndrome and provided evidence in support of genetic heterogeneity in this phenotype.

## Background

Microcornea is a congenital ocular disorder commonly reflecting abnormal development of the globe, especially of the anterior segment of the eye. The cornea in such patients demonstrate a horizontal diameter less than 10 mm even in adulthood, bilaterally [1, 2]. It can be associated with some other ophthalmologic and systemic symptoms, among which a rare dominantly inherited syndrome has been described and updated recently [3–6], comprising microcornea, retinal dystrophy, cataract, and posterior staphyloma (MRCS). In this MRCS pedigree, some disease-causing mutations have been subsequently identified in the gene BEST1 and ARL2 [7, 8]. However, no study has yet to report a coexistence of MRCS symptoms and exfoliation syndrome (XFS).

XFS is a genetically determined, age-related fibrillopathy which is characterized by ocular (and systemic) synthesis, accumulation and deposition of pathologic extracellular exfoliation material [9–11]. Exfoliation deposits are likely to infiltrate and alter iris and zonular lamella, leading to mydriatic-resistant pupil and zonule weakness [9]. Besides, the affected eyes are prone to secondary open-angle glaucoma as a result of the blockage of the trabecular outflow system by pigment granules and exfoliation material deposits. These ocular abnormalities may contribute to a more challenging cataract surgery and unexpected complications during perioperative management [12], let alone if accompanied with microcornea.

To our knowledge, combination of MRCS symptoms and XFS has not been previously reported, let alone associated with long axis. Here, we presented a case with MRCS syndrome, extremely long axial length and XFS, and revealed a possible novel candidate gene for MRCS syndrome.

## Case presentation

**Fig. 1:**
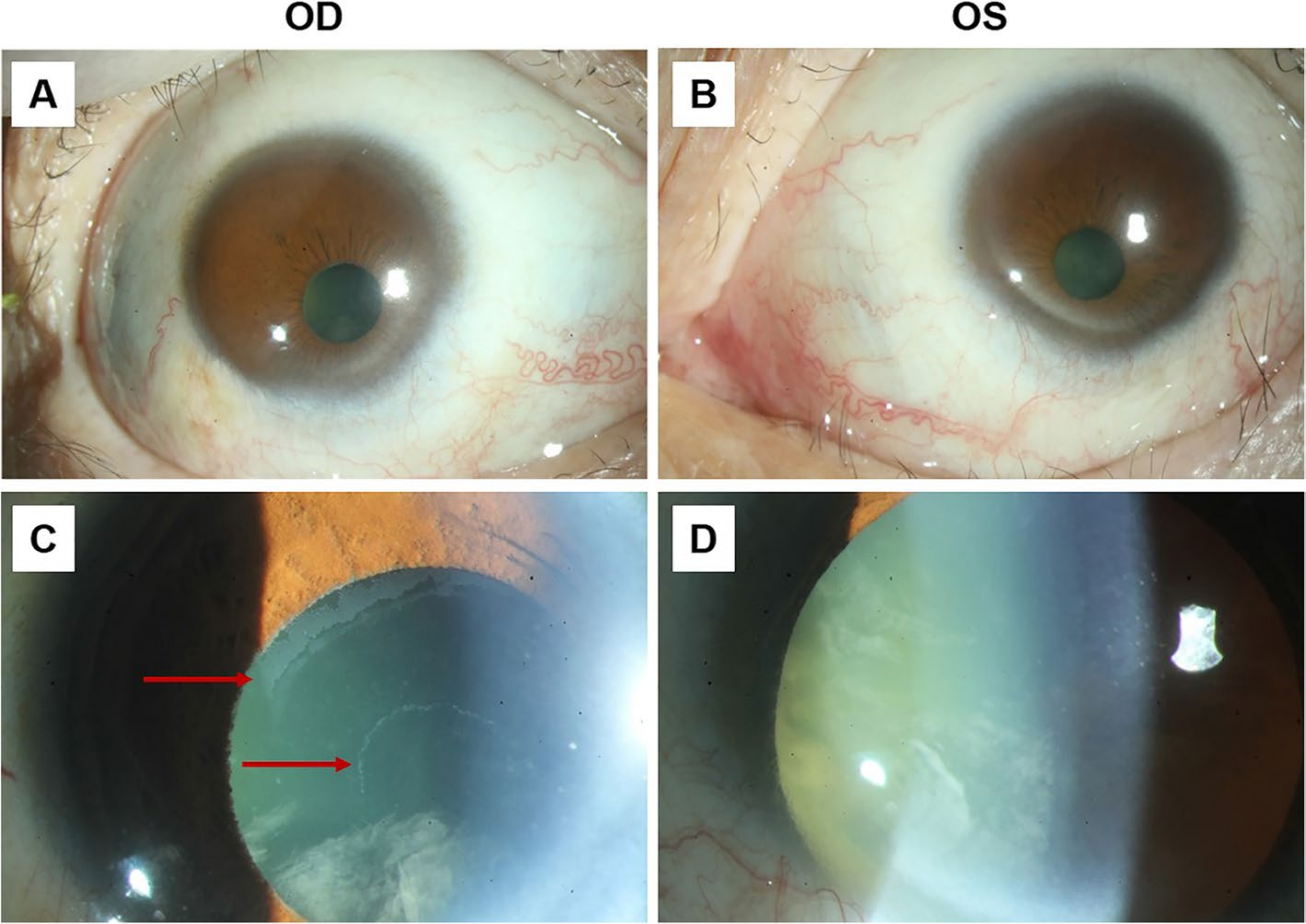
Preoperative photos of bilateral anterior segment. **A** and **B** The cornea was clear and the vertical and horizontal diameter was 7.5 mm and 8.0 mm on both sides. Her round-shaped, 3-mm-in-diameter pupil shifted towards inferior nasal direction. **C** Following pupillary dilation, ground-glass exfoliation deposits scattered on the anterior surface of the lens, around the edge of the pupil, with annular gray exfoliation deposits lined on the central anterior surface of the lens (red arrows). **D** No such deposition was identified in the left eye after pupillary dilation

**Fig. 2:**
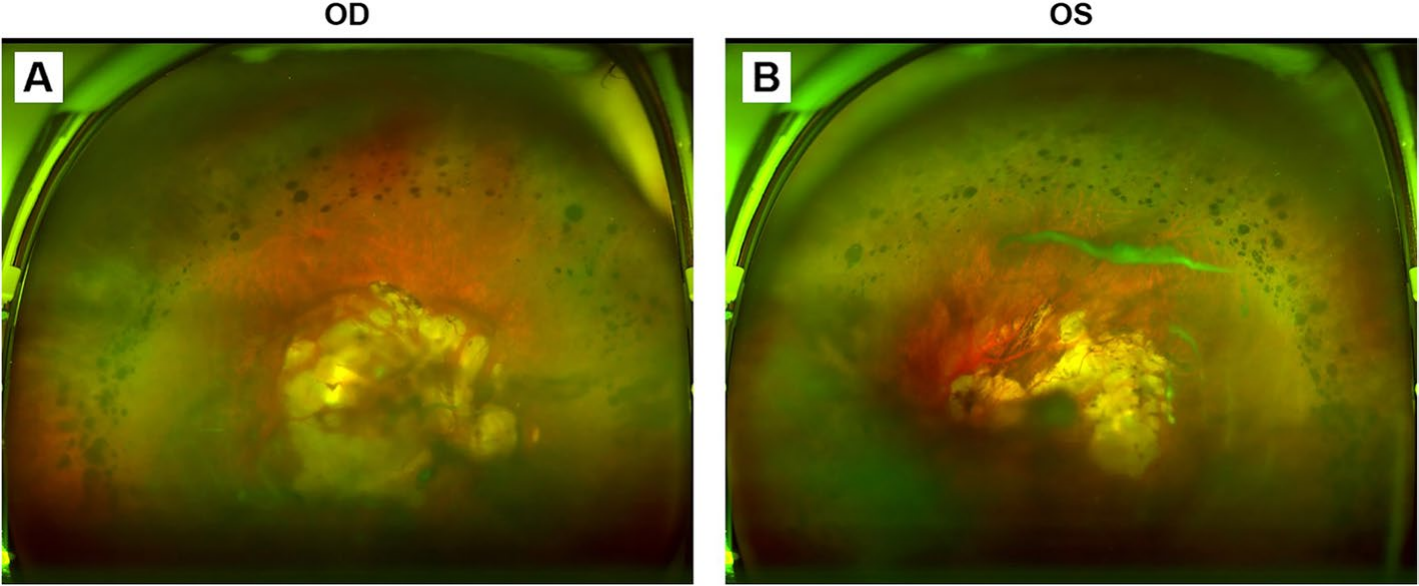
Wide-angle fundus photography of both eyes. **A** and **B** The detailed shape of optic disc was unrecognizable due to the posterior staphyloma in the background of tessellated fundus. Severe chorioretinal atrophy was present in the posterior pole and massive pigmentation scattered around the peripheral retina

A 66-year-old Han Chinese woman presented for consultation due to impaired vision which accompanied throughout her entire life span and worsened during the last 2 years. The patient suffered from poor eyesight in her childhood and started to wear eyeglasses since her teens with a best corrected visual acuity (BCVA) of 20/200 on both sides (the spherical equivalent of her glasses gradually declined from -8.0D to -18.0D over the years). Neither systemic nor ophthalmic disorder was present among her family members, including her late parents, 5 siblings and her son. On ophthalmic examination, her BCVA was finger count at 10 cm, with horizontal nystagmus in both eyes. Intraocular pressure (IOP) was 16 mmHg OD and 14 mmHg OS (Goldmann, Haag-Streit, Bern, Switzerland). The cornea was clear and the vertical and horizontal diameter was 7.5 mm and 8.0 mm on both sides, accordingly. Scattered flakes of fine clastic depositions were observed underneath the central area of corneal endothelium of the right eye (Fig. 1). In both eyes, the central anterior chamber depths were normal, with peripheral anterior chamber angle slightly narrowing (later confirmed by ultrasound biomicroscopy) (SUOER SW-3200L; Tianjin Sower Company, Tianjin, China) and no aqueous flare discerned. Her round-shaped, 3-mm-in-diameter pupil shifted towards inferior nasal direction and was circled with unclear textured iris. Following pupillary dilation, ground-glass exfoliation deposits became visible in the peripheral area on the anterior surface of the lens, around the edge of the pupil of the right eye, with annular gray exfoliation deposits lined on the central anterior surface of the lens. No such deposition was identified in the left eye. Distinct cortical and nuclear opacity could be spotted in both lenses. Gonioscopy revealed a wide open anterior chamber angle with wavy pigmented lines anterior to Schwalbe’s line and pigment aggregation in the meshwork of the right eye. The structure of bilateral vitreous body disclosed typical myopic posterior vitreous detachment which was further verified by ultrasonography. On dilated ophthalmoscopy, the detailed shape of optic disc was unrecognizable due to the posterior staphyloma in the background of tessellated fundus (Fig. 2). Severe chorioretinal atrophy was present in the posterior pole and massive pigmentation scattered around the peripheral retina. Non-contact specular microscopy (Topcon SP-3000, Topcon Optical, Japan) revealed a slightly below-average cell density of corneal endothelium in the right eye (cells/mm^2^, 2023; hexagonal cell percentage, 58%) while the corneal endothelium of her left eye was normal (cells/mm^2^, 2443; hexagonal cell percentage, 41%). Corneal thickness of both eyes were within normal range (561 μm OD and 550 μm OS). Her bilateral cornea both exhibited a regular front and back surface with an average refractive power of 47.11D OD and 48.26D OS. The anterior chamber depth was 2.79 mm OD and 2.53 mm OS. Extreme long axial length was detected in both eyes, specifically, 37.26 mm for the right eye and 34.29 mm for the left eye. All biometrics concerning the anterior segment was validated comparing data collected by ultrasound contact biometry, partial coherence interferometry (IOLmaster-700, Carl Zeiss Meditec, Jena, Germany) and topography (Pentacam, Oculus, Wetzlar, Germany). Measurements from IOLmaster-700 were selected and presented above. Optical coherence tomography failed to identify detailed structure of the retina and choroid due to the extreme long axis.

Since most of her clinical features fitted the description of a well-reported syndrome, MRCS, which was characterized by microcornea, retinal dystrophy, cataract (whereas XFS in our case), posterior staphyloma and mutations in the BEST1 (also known as VMD2) and ARL2 gene, a blood sample was drawn and genomic DNA was analyzed after informed consent was obtained. Mutation screening failed to identify disease-causing sequence variants in these specific genes. However, likely pathogenic mutations in FBN2 gene were identified as heterozygous variation. She was later diagnosed as extremely long axial length, MRCS and XFS, and was hospitalized for operations.

Following comprehensive and multidisciplinary pre-op evaluation (Table 1), bilateral cataract phacoemulsification without intraocular lens (IOL) implantation was performed under general anesthesia. Operation went successful and she was discharged two days later with no postoperative complication. At 3-month follow-up, her BCVA was 20/400 for both eyes and the IOP was 16 mmHg OD and 15 mmHg OS.

**Table 1:** Evaluation of the patient’s ocular condition, potential risk and corresponding perioperative management

Ocular condition	Potential risk	Perioperative management
**Microcornea**	• Limited operation sight and space	Scleral tunnel incision
	• Vulnerability to iatrogenic corneal damage during operation	
**Exfoliation syndrome (OD)**	• Poor pupil dilation	Preoperative topical nonsteroidal anti-inflammatory drugs in combination with mydriatic agents
	• Zonular weakness leading to potential lens subluxation and zonule dehiscence	No IOL implantation
**Extremely long axial length**	• Inaccuracy in IOL calculation;	
	• No power-wise appropriate IOL available;	
	• Beyond vitreoretinal surgery tool’s reach in case of capsule rapture during IOL implantation;	
	• Increased risk of massive suprachoroidal hemorrhage	General anesthesia

*IOL* Intraocular lens

## Discussion and conclusions

Microcornea has been proven to be a congenital abnormality which may associate with some other ophthalmologic and systemic symptoms, among which a well-described syndrome, MRCS, is comprised of microcornea, retinal dystrophy, cataract, and posterior staphyloma. However, no available research has reported a coexistence of MRCS syndrome, extremely long axis and XFS. Moreover, the failure to identify some previously-distinguished mutations in genomic sequence further suggests the genetic heterogeneity in subjects with a phenotype consistent with MRCS.

The clinical features of MRCS syndrome were comprehensively illustrated during the last two decades [3–7]. The affected subjects mostly displayed bilateral low vision due to cataract and macular dystrophy, which was in line with the case we presented here. One of the reasons that made this case unique is that previous MRCS patients, with the longest axial length in record of 26.40 mm [6], never had such a long axial length as the one we introduced here (34 mm plus, in both eyes) since microcornea frequently accompanies with diminution of entire anterior segment and occasionally the whole globe. Additionally, the displacement of pupils, which often accompanies with MRCS syndrome [13–15], was documented in this case as bilateral ectopic pupils shifting inferiorly and nasally for the first time. Based on her past medical history of low BCVA since early childhood, this prolonged ocular axis may result from a combination of form deprivation myopia and pathologic myopia. Genetic analysis in her pedigree was unfortunately impossible to complete because her parents already passed away and her 5 siblings were dispersedly settled across China and were unable to come to our research site due to the pandemic (Covid-19 and Omicron) as well as the corresponding restriction policy. We did manage to run autosomal and mitochondrial gene sequencing test on the patient and her only son, but mutation screening failed to identify disease-causing sequence variants in some specific genes reported previously, including BEST1 [4–6] and ARL2 [8]. However, likely pathogenic mutations in FBN2 gene were identified, likely providing novel perspective for future investigators and serve as a candidate gene for MRCS syndrome with proper subsequent experiment.

The concurrence of XFS in the patient’s right eye was evident even without pupil dilation, suggesting extensive accumulation of exfoliation material in the anterior segment. Deposition and infiltration of exfoliation material in the iris and zonular lamella drew us serious concerns on dealing with mydriatic-resistant pupil and zonule weakness in the following cataract surgery [16]. The potential IOP spikes during and after the surgery added more challenges in her perioperative management, even though her results from IOP testing and gonioscopy has temporally ruled out the diagnosis of exfoliative glaucoma [9]. After full evaluation of her ocular condition including MRCS syndrome, XFS and extremely long axis, bilateral cataract phacoemulsification without intraocular lens (IOL) implantation was performed using scleral tunnel incision and under general anesthesia. The adequate discussion and collaboration among different subspecialty ophthalmologists (experts in cataract, glaucoma and vitreoretinal disease contributed to the successful operation and patient’s satisfactory recovery.

To sum up, the case we presented here exhibited rare coexistence of MRCS syndrome, extremely long axial length and XFS. The complexity of her ocular abnormalities brought challenges to surgical management, yet the effective solution we adopted here could serve as an available option for future clinicians in case confronted with such patients. Furthermore, the genetic analysis in this case yielded a possible novel candidate gene for MRCS syndrome and provided evidence in support of genetic heterogeneity in this phenotype.

## Data Availability

All data and materials are available from the corresponding author at reasonable request.

## Abbreviations

ARL2: Adenosine diphosphate (ADP)-ribosylation factor-like 2
BCVA: Best corrected visual acuity
BEST1: Bestrophin 1
FBN2: Fibrillin 2
IOL: Intraocular lens
IOP: Intraocular pressure
MRCS: Microcornea, retinal dystrophy, cataract, and posterior staphyloma
VMD2: Vitelliform macular dystrophy 2
XFS: Exfoliation syndrome
